# Exopolysaccharides regulate calcium flow in cariogenic biofilms

**DOI:** 10.1371/journal.pone.0186256

**Published:** 2017-10-12

**Authors:** Monika Astasov-Frauenhoffer, Muth M. Varenganayil, Alan W. Decho, Tuomas Waltimo, Olivier Braissant

**Affiliations:** 1 Department of Preventive Dentistry and Oral Microbiology, University Center for Dental Medicine, University of Basel, Basel, Switzerland; 2 Department of Environmental Health Sciences, Arnold School of Public Health, Columbia, South Carolina, United States; 3 Center of Biomechanics & Biocalorimetry, c/o Department Biomedical Engineering (DBE), University of Basel, Allschwil, Switzerland; Oregon State University, UNITED STATES

## Abstract

Caries-associated biofilms induce loss of calcium from tooth surfaces in the presence of dietary carbohydrates. Exopolysaccharides (EPS) provide a matrix scaffold and an abundance of primary binding sites within biofilms. The role of EPS in binding calcium in cariogenic biofilms is only partially understood. Thus, the aim of the present study is to investigate the relationship between the calcium dissolution rates and calcium tolerance of caries-associated bacteria and yeast as well as to examine the properties of EPS to quantify its binding affinity for dissolved calcium. Calcium dissolution was measured by dissolution zones on Pikovskaya’s agar. Calcium tolerance was assessed by isothermal microcalorimetry (IMC) by adding CaCl_2_ to the bacterial cultures. Acid-base titration and Fourier transform infrared (FTIR) spectroscopy were used to identify possible functional groups responsible for calcium binding, which was assessed by isothermal titration calorimetry (ITC). *Lactobacillus* spp. and mutans streptococci demonstrated calcium dissolution in the presence of different carbohydrates. All strains that demonstrated high dissolution rates also revealed higher rates of calcium tolerance by IMC. In addition, acidic functional groups were predominantly identified as possible binding sites for calcium ions by acid-base titration and FTIR. Finally, ITC revealed EPS to have a higher binding affinity for calcium compared, for example, to lactic acid. In conclusion, this study illustrates the role of EPS in terms of the calcium tolerance of cariogenic microbiota by determining the ability of EPS to control free calcium concentrations within the biofilms as a self-regulating mode of action in the pathogenesis of dental caries.

## Introduction

Caries-associated biofilms cause loss of calcium and phosphate from tooth surfaces as bacteria living within such structures convert dietary carbohydrates into organic acids that diffuse into the tooth matrix and dissolve calcium phosphate at susceptible sites. Frequent exposure to fermentable carbohydrate leads not only to demineralization of dental hard tissues but also to compositional changes within the microbial community of the oral biofilm [1]. The vast majority of the early colonizers on dental surfaces belong to the “mitis group” of commensal streptococci (*Streptococcus sanguinis*, *Streptococcus oralis*, and *Streptococcus mitis*), whereas the pathogenic “mutans group” (*Streptococcus mutans*, *Streptococcus sobrinus*) comprises only 2% or less of the initial streptococcal population, regardless of the history of caries. In progressive stages of caries, however, up to 30% of the microflora in the lesions consists of mutans streptococci due to their acidogenic and aciduric properties compared to other streptococci [2]. Acidic conditions on the demineralized/decayed tooth surface serve as a selective factor enhancing virulent multispecies biofilm formation that is predominated by *S*. *mutans*, *Lactobacillus* species, aciduric strains of *Actinomyces* and oral yeasts [3].

In cariogenic biofilms, exopolysaccharides (EPS) provide an abundance of primary binding sites and form the core of the matrix-scaffold [3]. EPS consist of high-molecular weight polymers composed of insoluble complex-structured sugar residues such as glucans and/or fructans [4]. They are important in promoting the selective adherence and accumulation of large numbers of pathogenic streptococci in cariogenic lesions [3,5,6]. Furthermore, EPS increase the bulk and structural porosity of the biofilm, providing a highly structurally organized matrix including nutrition channels, thereby allowing more fermentable carbohydrate substrates to diffuse to the dental hard tissue surface, enhancing the development of progredient dental caries. EPS bear many different chemical functional groups such as carboxyl, phosphate and amine groups [7]. Among these groups, carboxylic acid and phosphates are known to bind calcium and other biologically important cations such as Mg^2+^, Fe^2+/3+^, and Zn^2+^ [8].

The role of EPS in binding calcium within cariogenic biofilms is important, as it is an essential regulatory ion for the vast majority of pro- and eukaryotic cells [9, 10], playing a key role in biofilm formation [11, 12, 13]. Different bacterial species have shown the ability to grow and form thick biofilms in the presence of naturally occurring levels of exogenous Ca^2+^ [12]. Within the human oral cavity, the Ca^2+^ concentrations of saliva and gingival crevicular fluid are generally 1.2–1.7 mM [11, 14]. Direct measurement of mineral release from the tooth surface has been applied for *in vitro*, *in situ*, and even *in vivo* samples [15, 16, 17]. However, the limitation of the methods is the presence of saliva or bacterial biofilms that can easily interfere with the analysis [18]. Thus, little is known regarding the concentration at which calcium and phosphate are dissolved from the tooth surface in a cariogenic lesion. Instead, it has been reported that released ions typically bind to salivary proteins or to bacterial cell walls [19]. However, when greater amounts of EPS are present in biofilms, fewer bacteria are detected, explaining the low concentrations of calcium found [20, 21]. Additionally, the profiles and concentrations of calcium-binding proteins in the oral cavity are strongly related to the carbohydrates present (e.g., differences in EPS composition have been identified when biofilms were formed in the absence of sucrose [20]). Furthermore, in cellular organisms, the cytoplasmic calcium concentration needs to be strongly regulated, usually remaining between 10 nM and 1 μM. Cytoplasmic calcium concentrations rising above 10 μM result in phosphate precipitation and thus compromise energy metabolism (mostly regulated by adenosine di- or triphosphate) [22]; therefore, many organisms have calcium efflux systems allowing them to tolerate calcium concentrations in the surrounding environment up to 1000 times higher [23].

The aim of the present study was to assess the ability of caries-associated bacteria and yeast to dissolve calcium from calcium phosphate and hydroxyapatite and to link such ability to the sensitivity of these strains to elevated calcium concentration. In addition, the acid-base and calcium binding properties of the EPS of selected strains were investigated. This study illustrates the relationship between calcium dissolution and calcium tolerance as well as the role of EPS in high calcium concentration tolerance.

## Materials and methods

### Strains

Type strains of the following caries-associated bacteria and yeast were used in this study: *Streptococcus sobrinus* (ATCC 33402), *Streprococcus mutans* (ATCC 25175), *Lactobacillus paracasei* (DSM 20020), *Lactobacillus casei* (DSM 20011), *Actinomyces viscosus* (ATCC 43146), *Aggregatibacter actinomycetemcomitans* (DSM 8324) and *Candida albicans* (ATCC 90028). Additionally, clinical isolates (collected and identified during routine screening for different infections) of *S*. *sobrinus*, *S*. *mutans*, *Lactobacillus paracasei*, *Lactobacillus rhamnosus*, and *C*. *albicans* as well as two non-caries related control strains of *Escherichia coli* (ATCC 25922) and *Staphylococcus epidermidis* (ATCC 49461) were tested. All strains were inoculated in 5 ml of Luria broth (BBL^™^, Becton Dickinson, Basel, Switzerland) supplemented with glucose (10 g/l; Sigma-Aldrich, Buchs, Switzerland) and incubated for 24 h at 37°C under aerobic conditions (except for *A*. *actinomycetemcomitans*, which was incubated in MACS MG (Don Whitley Scientific Ltd Meintrup DWS Laborgeräte GmbH, Herzlake, Germany; atmosphere of 80% N_2_, 10% H_2_ and 10% CO_2_). Stock solutions of strains were prepared in 20% glycerol (Sigma, Buchs, Switzerland) and stored at -70°C.

### Calcium dissolution

To assess dissolution of calcium phosphate, the cultures were grown on Luria broth agar (BBL^™^, Becton Dickinson, Basel, Switzerland) supplemented with glucose (10 g/l; Sigma-Aldrich, Buchs, Switzerland) at 37°C for 24 h under aerobic conditions, and the resulting colonies were inoculated into one of the three types of Pikovskaya’s agar [24] supplemented with Ca_3_(PO_4_)_2_ (Sigma-Aldrich, Buchs, Switzerland), Ca_5_(PO_4_)_3_OH (particle size <1 μm), or Ca_5_(PO_4_)_3_OH (particle size <15 μm) (Medipure powder, Medicoat, France) and one of the following four sugars: glucose, sucrose, fructose, or lactose (10 g/l; Sigma-Aldrich, Buchs, Switzerland). Plates were incubated for 1 week at 37°C under aerobic conditions before the dissolution zones (mm) were measured, n = 10.

### Calcium toxicity

Cultures were suspended in Luria broth supplemented with glucose (10 g/l) and containing one of five different concentrations (1, 3, 10, 30, or 100 mmol/l) of CaCl_2_ (Sigma-Aldrich, Buchs, Switzerland). Growth of the samples was monitored by isothermal microcalorimetry (IMC;calScreener^™^, SymCel Sverige AB, Sweden) over 6 d at 37°C. The maximum growth rate (1/h) of the samples was calculated from the heat flow data collected over time using an exponential growth rate model [25] in R software [26], n = 4.

### Exopolysaccharide (EPS) extraction

The clinical isolates of *S*. *mutans CI2*, *L*. *rhamnosus CI*, and *C*. *albicans CI1* that showed the highest resistance to Ca^2+^ ions in the calcium toxicity measurements were chosen for EPS extraction. From a stock solution of the strain, 100 μl was inoculated into 5 ml Luria broth (BBL^™^, Becton Dickinson, Basel, Switzerland) supplemented with glucose (10 g/l; Sigma-Aldrich, Buchs, Switzerland) and then incubated for 24 h at 37°C under aerobic conditions. Thereafter, 1 ml of preculture was inoculated into 1 l of 50% Luria broth + glucose medium and incubated 48 h at 37°C under aerobic conditions. Subsequently, the cultures were processed through glass-fiber filters (Millipore^®^ Type 2 filters, retention 1.0 μm; Sigma-Aldrich, Buchs, Switzerland) and 0.22-μm filters (Millipore^®^ Stericup^™^ filter units PVDF membrane (Durapore); Sigma-Aldrich, Buchs, Switzerland). The filtrate was recovered, and 2 parts cold ethanol (Sigma-Aldrich, Buchs, Switzerland) (-20°C) per 1 part filtrate (v/v) was added. The mixture was briefly stirred and then stored at 4°C overnight to allow the EPS to precipitate. The solution was centrifuged at 3000 rpm (ROTINA 380 R, Hettich Zentrifugen, Tuttlingen, Germany) for 10 min at 4°C, and the recovered EPS pellet was dissolved in cold ethanol before being placed in dialysis bags (cellulose membrane, molecular weight cut-off 14000 Da; Sigma-Aldrich, Buchs, Switzerland). The EPS was dialyzed 2x against 1 mM EDTA (Sigma-Aldrich, Buchs, Switzerland), followed by 3x in ultrapure water at 4°C. The dialyzed EPS was stored at 4°C. To determine the concentration of the purified EPS, 7.5 ml of the final solution was lyophilized (Integrated SpeedVac System ISS110, Savant, Fischer Scientific AG, Reinach, Switzerland) and weighed.

### Acid-base titration of EPS

Proton-binding sites present in EPS were determined by acid-base titration. Fifteen milliliters of dialyzed EPS (approx. 10 mg) was added to 15 ml of ultrapure water, and 30 μl of 1 mM KCl (Sigma-Aldrich, Buchs, Switzerland) was added to adjust ionic strength. The solution was analyzed under a constant flow of nitrogen (99.9%, Pangas, Pratteln, Switzerland) to prevent CO_2_ dissolution and formation of carbonate ions. The initial pH of the solution was adjusted to 2.6 and was titrated with 0.1 N NaOH (Sigma-Aldrich, Buchs, Switzerland). The pH was recorded during titrations with an Orion PerpHect LogR 330 pH meter (Orion, Boston, MA) until a pH of 10.5 was reached. The titration curves obtained were analyzed using PROTOFIT 2.1 software. For the analysis, a three-site, non-electrostatic model as recommended for biological surfaces was assumed [27]. The results were verified by FTIR analysis.

### Calcium binding with isothermal titration calorimetry (ITC)

Calcium-binding properties were investigated using ITC. The adsorption enthalpy of Ca^2+^ on EPS was measured in a TAM III calorimeter (TA instruments) equipped with a micro-reaction system with a 3-ml stainless steel ampoule. The ampoule was lowered into the calorimeter using a four-step thermal equilibration procedure. After these steps, the ampoule was placed in the measuring position, and the titration was initiated when the signal stability was close to 200 nW per 30 min. The solution in the ampoule contained purified EPS (see above sections) combined with Tris buffer to reach a final concentration of 20 mM Tris. A 250-μl Hamilton syringe was filled with a solution containing 20 mM Ca^2+^ and 20 mM Tris. The syringe capillary was inserted into the titration unit until the needle tip was submerged in the solution in the ampoule. The titrations consisted of 25 injections of 10 μl solution each. The resulting heat flow signal was collected and analyzed using the TAM assistant software assuming only one binding site. Blank titrations (i.e., 20 mM calcium solution in Tris buffer injected in Tris buffer only) were performed to subtract the non-specific signal. Three replicate runs for the two bacteria strains, two runs for *Candida*.

### Fourier transform infrared (FTIR) spectroscopy

Fourier transform infrared spectroscopy (FTIR) was used to determine the presence of specific functional groups including carboxyl, sulfate, sulfinic acid, thiol, hydroxyl and amino groups on EPS (37). Analyses were conducted on a Nexus 670 FTIR spectrometer (Thermo-Nicolet Inc., Waltham, MA, USA) equipped with attenuated total reflectance (ATR) and fitted with a multibounce germanium crystal (Thermo-Nicolet Inc.). Dry EPS samples (approx. 1 mg) were placed in a Thunderdome Tilt-back Pressure Tower (Spectro-Tech Foundation Series, Nicolet, Madison, WI, USA), in order to facilitate optimal contact between the sample and the crystal. Absorbance spectra were collected between 4000 and 600 cm^–1^ at a spectral resolution of 4 cm^–1^ or 8 cm^–1^, with 64 scans co-added and averaged. If necessary, baseline corrections were carried out.

### Statistical analysis

The data from calcium dissolution measurement were first analyzed for normal distribution using the Shapiro-Wilk test. All data were found normally distributed; hence, one-way ANOVA was chosen to test for differences between species in calcium dilution as well as calcium tolerance. Student’s t-test was applied to find differences between caries related and non-cariogenic control species in calcium dissolution. Statistical significance was set at p < 0.05.

## Results

### Calcium dissolution

**Fig 1:**
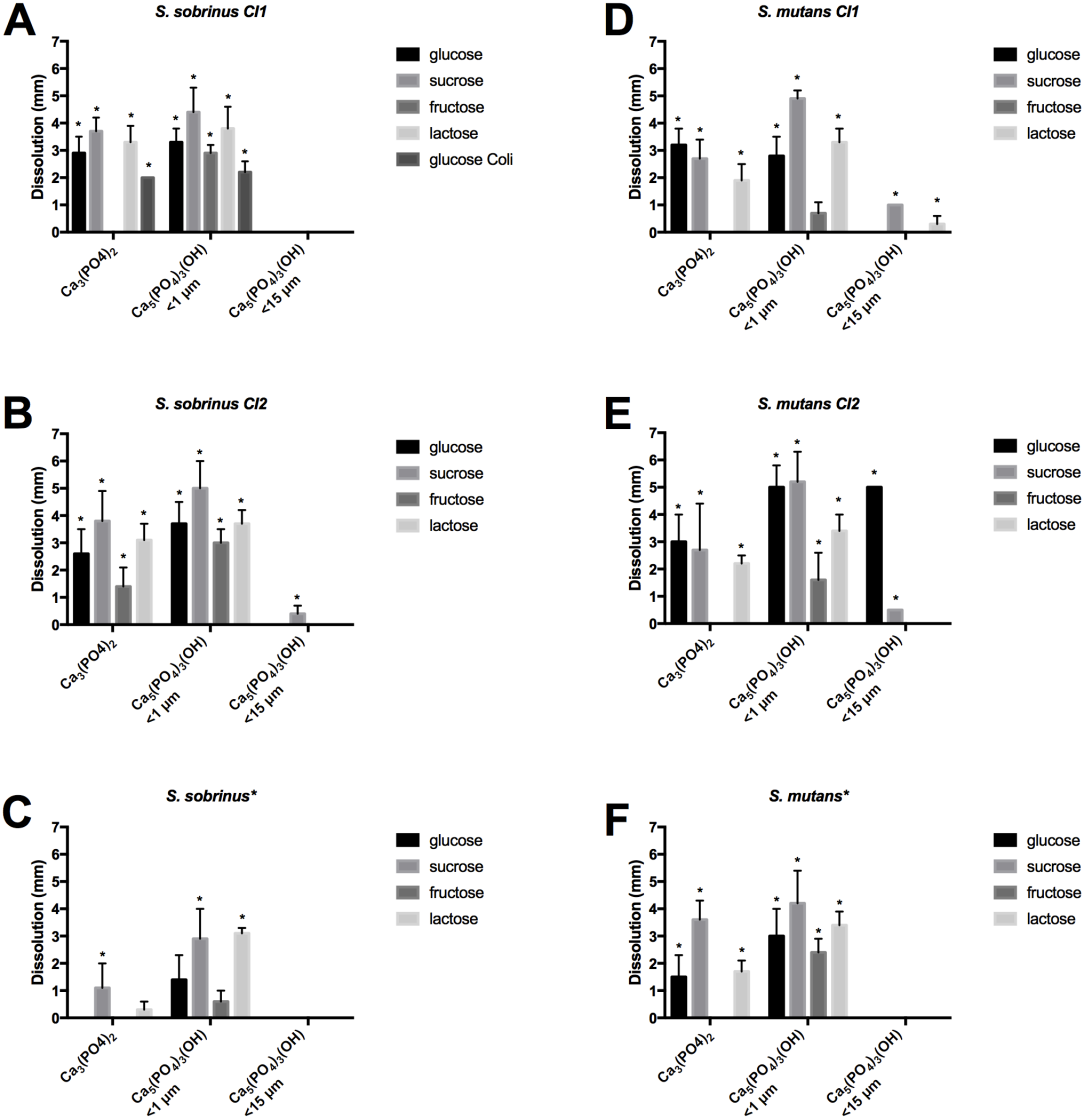
Calcium dissolution by caries-associated mutans streptococci (*S*. *sobrinus* (A—C) and *S*. *mutans* (E—F)) was investigated by measuring dissolution zones (mm; mean ± SD) on Pikovskaya’s agar in the presence of four different sugars. Asterisks next to the name mark type strains; CI indicates clinical isolates used. Statistically significant differences (p < 0.05) are noted with an asterisk. No dissolution was detected for *A*. *actinomycetemcomitans* or *Candida* spp. N = 10.

**Fig 2:**
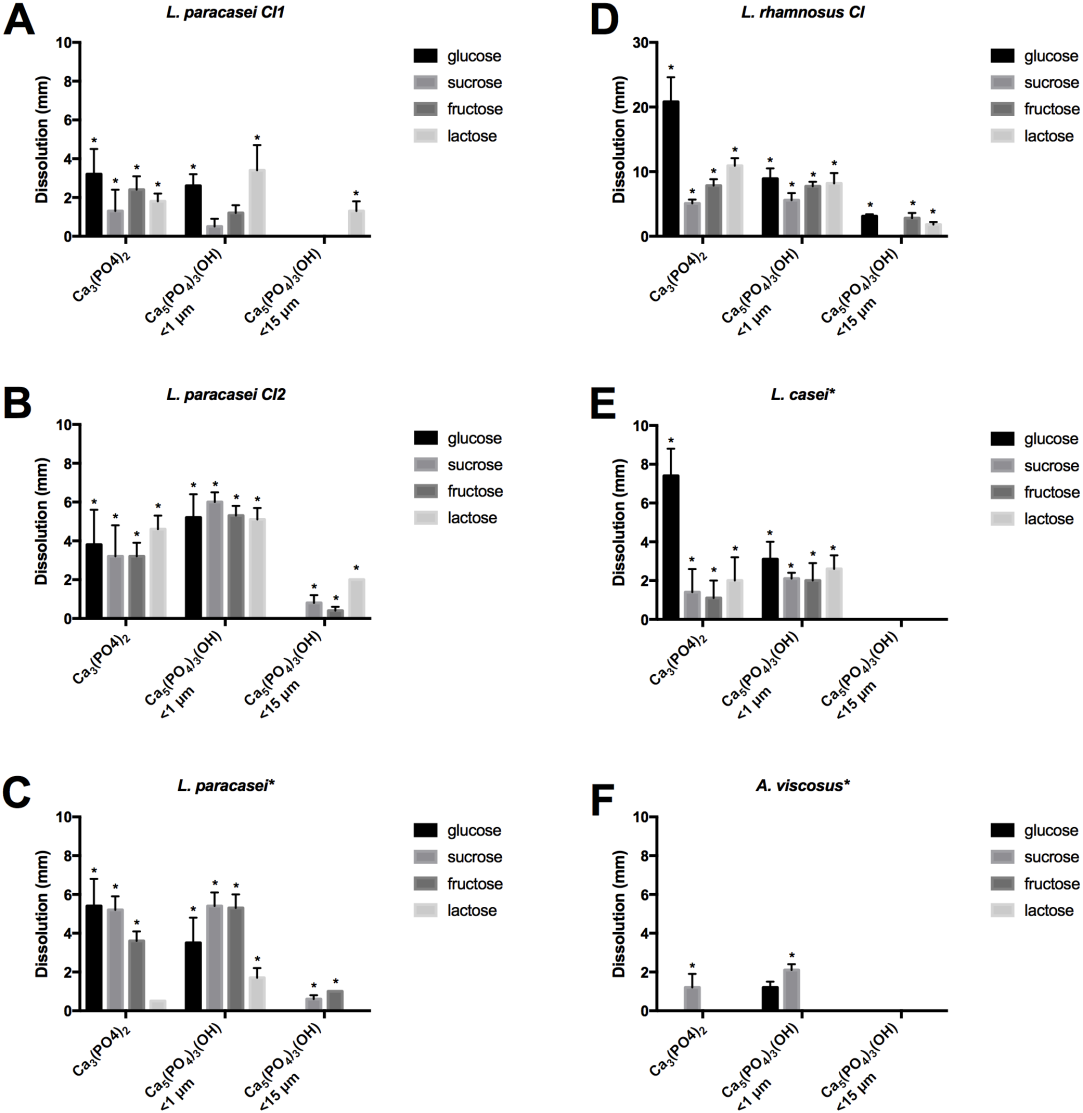
Calcium dissolution by caries-associated lactobacilli (A—E) and actinomycetes (F) investigated by measuring dissolution zones (mm; mean ± SD) on Pikovskaya’s agar in the presence of four different sugars. Asterisks mark type strains; CI indicates clinical isolates used. Statistically significant differences to non-caries related strains (p < 0.05) are noted with an asterisk. No dissolution was detected for *A*. *actinomycetemcomitans* or *Candida* spp. N = 10.

Calcium dissolution was investigated by using four different dietary sugars (glucose, sucrose, fructose, and lactose) and three different types of calcium phosphate (Ca_3_(PO_4_)_2_ and fine- and rough- grained Ca_5_(PO_4_)_3_(OH)). All bacterial strains except for *A*. *actinomycetemcomitans* and *Candida* spp. showed dissolution of at least one of the three forms of calcium phosphate added to the medium (Figs 1 and 2).

Most of the other strains showed a significantly higher dissolution potential (p < 0.05, n = 10) of at least one type of calcium phosphate compared to non-cariogenic strains (*E*. *coli* and *S*. *epidermidis*) when an ANOVA test was performed. Additionally Student’s t-test was performed to assess where the differences were significant (p < 0.05, n = 9). *Lactobacillus* strains were able to dissolve hydroxyapatite with higher efficacy than mutans streptococci for all sugars tested. Interestingly, one clinical isolate (*L*. *rhamnosus*) dissolved some forms of calcium phosphate up to five times more efficiently than others strains in this study (Fig 2). The type strain of *A*. *viscosus* demonstrated only moderate dissolution that did not significantly differ from that of the non-cariogenic control species (p > 0.05) (only two sample groups showed significant dissolution).

### Calcium toxicity

**Fig 3:**
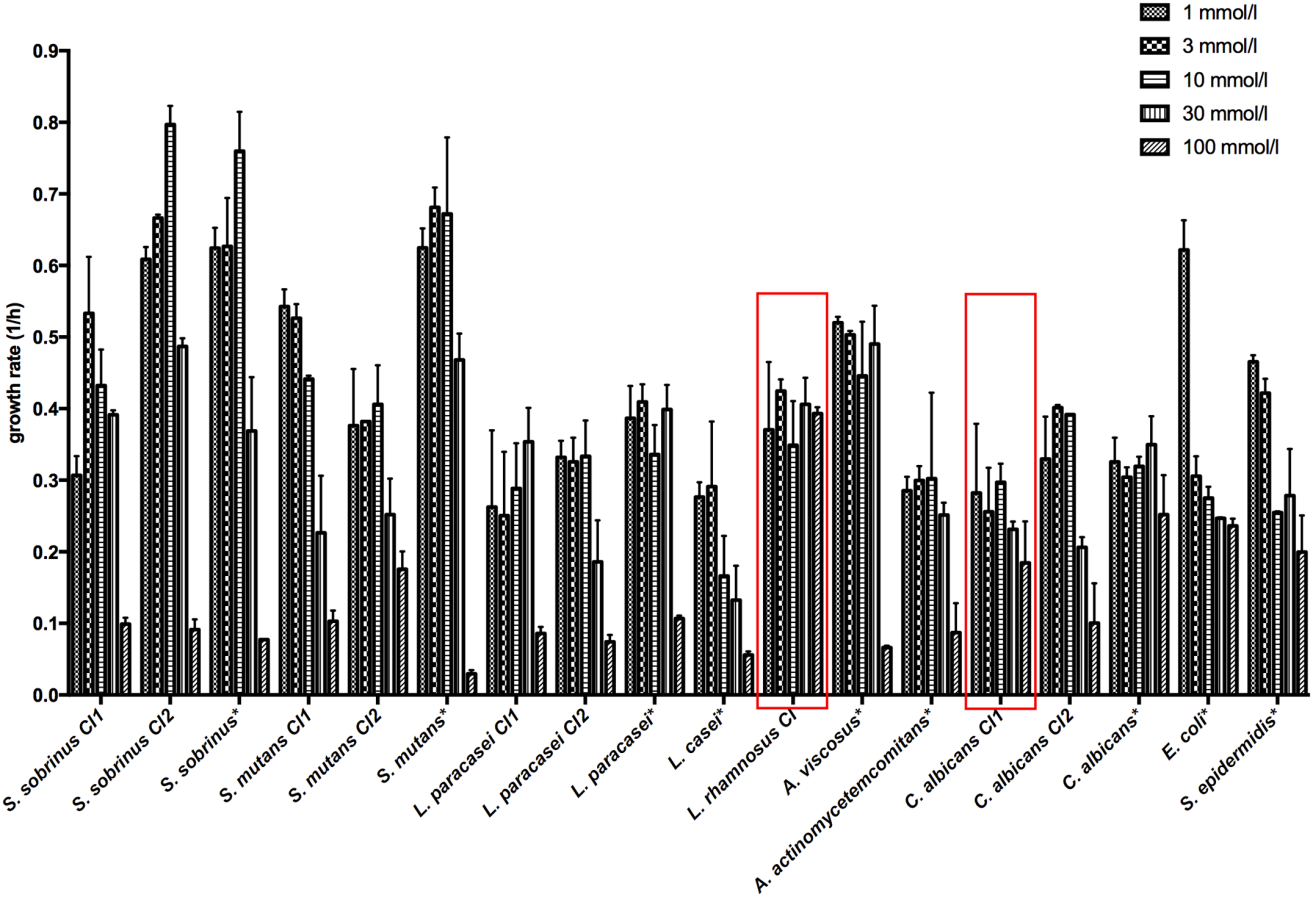
Effects of Ca^2+^ ions in different concentrations on the growth rates of the cariogenic species (mean ± SD). N = 4. *L*. *rhamnosus CI* and *C*. *albicans CI1* are highlighted as strains showing no significant decrease in growth rate when exposed to 100 mM CaCl_2_ (p <0.05).

The growth rate of most bacterial and fungal organisms was affected by the concentration of calcium added to the medium. For both non-cariogenic strains, the calcium tolerance was relatively low, and the highest growth rate was observed at a CaCl_2_ concentration of 1 mM. The growth rate at this concentration was particularly high for *E*. *coli* when compared to other concentrations (Fig 3).

Many of the cariogenic strains exhibited optimum growth between 3 and 30 mM, which is already approximately 2- to 20-fold higher than what is typically encountered in saliva. Only *L*. *rhamnosus CI* and *C*. *albicans CI1* were able to withstand 100 mM without a significant decrease in growth rate (p < 0.05). Additionally, *L*. *rhamnosus CI* was able to withstand a concentration of 300 mM (data not shown) with only a minor decrease in growth rate (up to 20%).

### Acid-base characterization of EPS

Three of the tested microorganisms from different species (*S*. *mutans CI2*, *L*. *rhamnosus CI*, *and C*. *albicans CI1*) were selected for further characterization of their EPS based on their calcium dissolution and calcium tolerance. From a quantitative point of view, the two bacterial strains produced higher amounts of EPS compared to *C*. *albicans CI2*. Acid-base titration of the EPS revealed that mostly acidic proton binding sites were present (Table 1); the pK values of these sites were between 2.0 and 5.3. Binding sites with such pK values usually feature carboxyl groups (i.e., -COOH). Some binding sites with near-neutral and alkaline pH were observed, but in much lower concentrations. Sites with pK values between 6.0 and 7.0 usually feature phosphate groups (i.e., -O-PO_3_H). These results were verified by FTIR analysis [28].

**Table 1:** Estimated dissociation pH values for various functional groups present in EPS samples obtained by acid-base titration after EPS was precipitated with ethanol or propanol.

Strain	Yield (mg/ml)	Site 1		Site 2		Site 3	
		pH	Conc. (mol/kg)	pH	Conc. (mol/kg)	pH	Conc. (mol/kg)
*S*. *mutans CI2*	0.72–0.75	2.0–2.1	1.40–1.50	5.2–6.7	0.16–0.20	>6.9	0.30–0.39
*L*. *rhamnosus CI*	0.66–0.68	2.6–4.5	0.40–0.75	5.1–6.2	0.20–0.50	>7.00	0.15
*C*. *albicans CI1*	0.65–0.69	2.5–3.6	0.30–1.00	5.1 5.3	0.15–0.40	>7.02	0.18

### FTIR results

Major peaks in the FTIR spectra confirmed the presence of carbohydrates (C-O from carbohydrate stretch: 1037, 1058 cm^-1^) and carboxylic acid (C = O; 1637 cm^-1^, 1653 cm^-1^, 1666 cm^-1^, 1684 cm^-1^, 1699, cm^-1^) in all EPS tested. The absence of buffering due to ammonium or amine at alkaline pH (see Table 1) suggests that the bands at 1666 cm^-1^ and 1684 cm^-1^ found in *C*. *albicans CI1* could also indicate that the presence of amino groups is unlikely. All EPS-extracted peaks at 2340 cm^-1^ and 2360 cm^-1^ could be attributed to O-H doublets from sulfinic or sulfonic acids, to P = O stretches from phosphates, or to C = N bonds. Considering once more the pH buffering observed, phosphate groups or sulfur compounds seem most likely to be present. Finally, all EPS showed a small peak at 2950 cm^-1^ that was attributed to -OH or -NH groups.

In addition to the peaks common to all EPS, other peaks appeared. Peaks at 1404 cm^-1^ and 1415 cm^-1^ were attributed to carboxylate in *L*. *rhamnosus CI* and *C*. *albicans CI1*. Peaks at 1538 cm^-1^ and 1541 cm^-1^ were attributed to amides and indicated that some peptide moieties might still be present in the EPS from in *S*. *mutans CI2* and *C*. *albicans CI1*. Peaks at 740 cm^-1^ and 743 cm^-1^ were attributed to alcohol groups in *S*. *mutans* and *C*. *albicans*. Finally, ester bonds were seen in *L*. *rhamnosus CI* EPS, making a peak at 1223 cm^-1^.

### Calcium binding to EPS

Isothermal titration calorimetry (ITC) showed that EPS from bacterial strains could bind calcium more efficiently than lactic or citric acid (Table 2). The binding affinity (k) was one order of magnitude higher than those of the organic acids. However, the calcium binding affinity of EPS was still low compared to strong chelating agents such as EDTA (Table 2). Values for ΔH and ΔG suggest largely electrostatic binding mechanisms, which also result from the presence of carboxylic acids on the EPS. For EPS purified from *C*. *albicans CI1*, titration data revealed relatively weak binding that did not allow an accurate estimation of binding affinity. Thus, only ΔH was calculated, and the other thermodynamic parameters were not evaluated. Based on these results and the total heat released during the titration, the binding capacities were estimated to be 25 mg_ca_/g_EPS_ for *L*. *rhamnosus CI*, 9 mg_ca_/g_EPS_ for *C*. *albicans CI2* and 16 mg_ca_/g_EPS_ for *S*. *mutans CI2*.

**Table 2:** Thermodynamics of Ca^2+^ binding properties to EPS produced by caries-associated species used in this study and to some reference compounds.

Strain	k	ΔH [kJ*mol^-1^]	ΔG [kJ*mol^-1^]	ΔS [J*kg^-1^*mol^-1^]	n
*S*. *mutans CI2*	2.2*10^4^ ± 6.3*10^3^	30.5 ± 0.5	-26.5 ± 1.4	165.5 ± 27.4	3
*L*. *rhamnosus CI*	2.1*10^4^ ± 3.2*10^3^	20.1 ± 2.6	-25.7 ± 0.3	148.0 ± 9.8	3
*C*. *albicans CI1*	Weak (<10^3^)	35.2**	ND	ND	2
EPS	ND	24.11	-12.24	121.98	
EDTA *	1*10^10^				
Citric acid *	3.1*10^3^				
Lactic acid *	1.6*10^3^				

* Added for comparison purposes.

** Data to be taken with care.

ND: not determined.

## Discussion

This study illustrates variations in the calcium phosphate dissolution potential and calcium tolerance of caries-associated microbial strains. These data are in accordance with the three-step dental plaque model of Takahashi and Nyvad [2] that shows the relations between the oral ecosystem and the dominance of different microorganisms in three different phases. As long as natural pH is maintained, the key organisms needed for the dynamic stability stage, such as non-mutans bacteria (mainly non-mutans streptococci and *Actinomyces* species), are found in high percentages. At this stage, the caries lesions are not formed, and the free calcium concentration around the site is probably close to equilibrium with the concentration in saliva (i.e., 1.2–1.7 mM) [11, 14].

When the pH is lowered by cariogenic species and caries develops, these non-mutans bacteria are challenged to survive in an acidic environment. Once the acidogenic phase has been reached, the environment change promotes a bacterial species shift from non-mutans to mutans streptococci and other aciduric species (i.e., *Lactobacillus* spp., opportunistic periodontal pathogens such as *A*. *actinomycetemcomitans*, and *C*. *albicans*). These microorganisms promote caries lesion development with their metabolism. Acidogenic mutans streptococci are able to form extracellular polysaccharides (EPS) in the presence of sucrose, fructose and glucose [29, 30]. At this stage, the cariogenic biofilm is exposed to higher calcium concentrations, as calcium release from the tooth surface occurs in a semi-closed environment (i.e., the caries lesion and its surrounding biofilm). Such high calcium concentrations, in turn, might have a negative effect on microorganisms [22]. However, in this study, most of the strains of *Streptococcus* and *Lactobacillus* show higher calcium tolerance than other caries-related stains. Indeed, many of them grew faster at calcium concentrations ranging from 3 mM to 30 mM. As lactate is often converted into other organic acids by cariogenic bacteria, it cannot further bind the calcium released from the teeth. However, within the biofilm, matrix EPS can bind calcium with a higher affinity than lactate, as shown in this study. This provides bacteria with a possible mechanism to withstand higher concentrations of calcium as they integrate it to their biofilms, thereby neutralizing the toxic effects.

Similarly, *Lactobacillus* spp. are well-known as EPS producers from food microbiology studies [30–32]. *Lactobacillus* spp. and mutans streptococci strains showed comparable results in calcium dissolution. Overall, the clinical isolates in this study showed a slightly higher dissolution and calcium affinity in this study. One studied strain, the clinical isolate of *L*. *rhamnosus*, demonstrated efficacy up to five-fold higher than any other species investigated in calcium dissolution, and its growth rate was not affected by high calcium ion concentration, illustrating thereby the main hypothesis of the study, that cariogenic species adapt to conditions in the lesion by finding mechanisms to overcome them, and one of those mechanisms is calcium binding by EPS.

For bacteria, a correlation between the capacity for solubilizing calcium and tolerance to elevated calcium was observed (*r* = 0.72, *n* = 13, *p* = 0.002). This emphasizes that calcium tolerance might play a crucial role in cariogenic bacteria. *Candida* species were excluded from the correlation because as eukaryotic cells, yeasts have other mechanisms to overcome the toxic effects of calcium cations. Indeed, yeasts are known to intracellularly store excess calcium in vacuoles [33]. Such a mechanism might explain the opportunistic presence of *Candida* spp. in caries even if they are not the main cariogenic agent (strains tested in this study were unable to dissolve calcium phosphate but could withstand high extracellular calcium concentration). The levels of calcium are maintained through transient receptor potential channels, including selective and nonselective cation-permeable ion channels [34]. These channels predominately mediate calcium transport from the extracellular environment into the cell or from intracellular calcium stores to the cytoplasmic matrix, controlling the free cytoplasmic calcium levels [35, 36].

Although this study provides additional data regarding the potential role of calcium tolerance and EPS calcium binding properties in mediating calcium effects on microbial cells in caries lesions, there are some limitations to the methods. The use of glucose as a “generalistic” substrate might be appropriate for some strains based on the data presented in this study (*L*. *rhamnosus* and *L*. *casei*). However, EPS production in other strains (such as *S*. *mutans*) clearly benefits from using sucrose as the substrate for EPS production. Overall, to get a complete picture of calcium tolerance and binding by cariogenic bacteria, the measurements performed here with glucose should be repeated and validated with other carbon sources such as sucrose and fructose.

Another limitation is the fact that the calcium-binding capacity of the EPS might be underestimated by ITC. Indeed, ITC measures the changes in enthalpy associated with ligand binding; however, it is possible that the binding of additional Ca^2+^ could be entropically (rather than enthalpically) driven. As ITC is a technique that is well fitted for measuring medium- to high-affinity binding, additional low-affinity binding sites might be masked by the heat of dilution of the ligand (CaCl_2_) into the EPS solution. Additionally, many neutral oxygen molecules (from amide bonds or ester bonds detected in the FTIR analysis) could, for example, be involved in weak binding of Ca^2+^. As a result, low binding capacities of 25 mg_ca_/g_EPS_ for *L*. *rhamnosus CI*, 9 mg_ca_/g_EPS_ for *C*. *albicans CI1* and 16 mg_ca_/g_EPS_ for *S*. *mutans CI2* were observed, compared to studies using ion-specific electrodes, in which values up to 150 mg_ca_/g_EPS_ have been measured for EPS produced by an environmental strain [37]. That, however, is in accordance with titration data showing more possible binding sites.

## Conclusions

**Fig 4:**
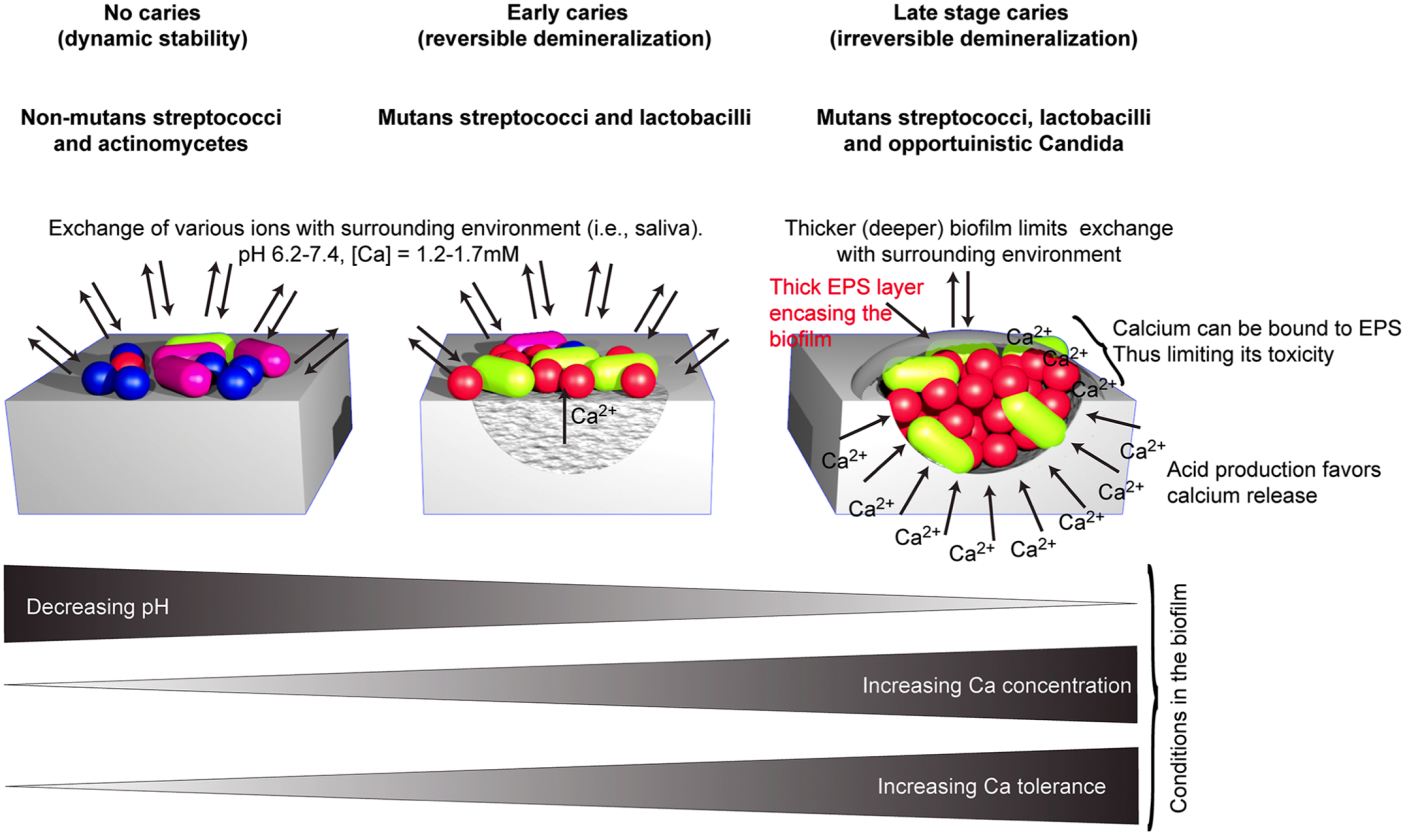
A simplified model illustrating the flow of calcium during different stages of caries formation and its binding to EPS.

In conclusion, this study illustrates the relationship between calcium dissolution and calcium tolerance as well as the role of EPS in high oral calcium concentration tolerance (Fig 4). Moreover, the study reveals the novel finding that cariogenic species have a regulation mechanism to eliminate possibly toxic amounts of calcium ions by binding them to strengthen their own biofilm core, and at the same time, affecting the rates of free calcium for remineralization to their favor.
